# A case of skin necrosis following pethidine intravenous injection

**DOI:** 10.1093/jscr/rjad431

**Published:** 2023-07-29

**Authors:** Abdullh AlQhtani

**Affiliations:** Plastic Surgery, Surgery Department, College of Medicine, Prince Sattam Bin Abdulaziz University, Al Kharj 16273-7201, Saudi Arabia

## Abstract

Pethidine is an opioid derivative typically used to treat moderate-to-severe pain. It has a good analgesic effect and is considered safe, although with adverse effects such as nausea, depression, vomiting, respiratory depression, and toxicity. We discuss the case of a 43-year-old female patient who received intravenous pethidine and developed skin necrosis in multiple areas of the body as a complication and how it was managed. This is the first report in the literature discussing skin necrosis after intravenous pethidine.

## INTRODUCTION

Pethidine is an opioid derivative that was synthesized in 1939 in Germany [1]. It is the first synthetic drug with the potential to relieve pain. Meperidine is one class of phenylpiperidines [2–5] usually used for the treatment of moderate-to-severe pain and the reduction of shivering. Although it has a good analgesic effect and is considered safer than other opioids due to its lower risk of addiction because of its anticholinergic effects, there is a high incidence of adverse effects such as nausea, depression, vomiting, and respiratory depression due to its toxic metabolites [6].

Here, we discuss the first reported case of skin necrosis in multiple areas of a patient’s body after receiving IV pethidine.

## CASE REPORT

**Figure 1:**
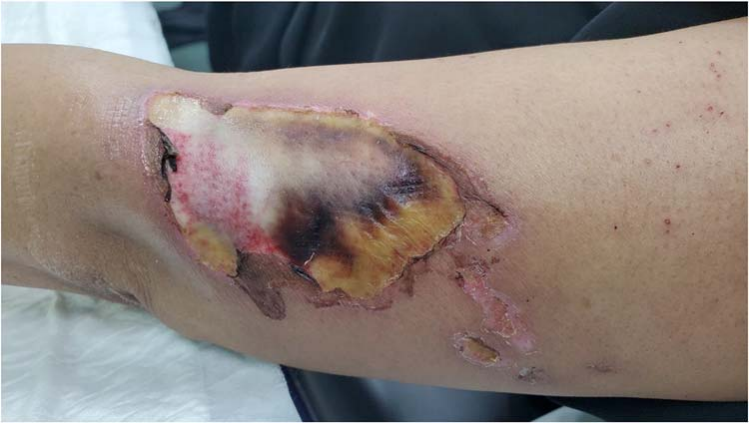
Wounds on the left arm when the patient presented to our clinic 2 weeks post pethidine Intravenous Injection.

A 43-year-old female patient presented to our clinic with wounds on the left arm, right thigh, and upper helix of the right ear (Fig. 1).

The patient had no known comorbidities, was not on any regular medications, and had a family history of diabetes mellitus II and hypertension.

She had undergone two cervical spine surgeries in 2017 and 2019 and visited the emergency room two weeks before for severe pain in the neck. She received intravenous pethidine 50 mg on the dorsum of the left hand, after which a wound started to appear on her left arm, right thigh, and upper helix of the right ear.

On examination, the patient’s vital signs were stable. She developed a deep burn wound on the lateral side of her left arm (approximately 12x7 cm), a small wound on the upper helix of her right ear (approximately 0.5 cm) which was starting to heal, and a deep burn wound on the right thigh (approximately 1x1 cm) which was starting to heal from the periphery.

None of the wounds were infected or dressed daily. After one week, the thigh and ear wounds healed, but debridement was required in the left arm. Debridement was performed and primary closure was possible due to the redundant skin on the arm.

**Figure 2:**
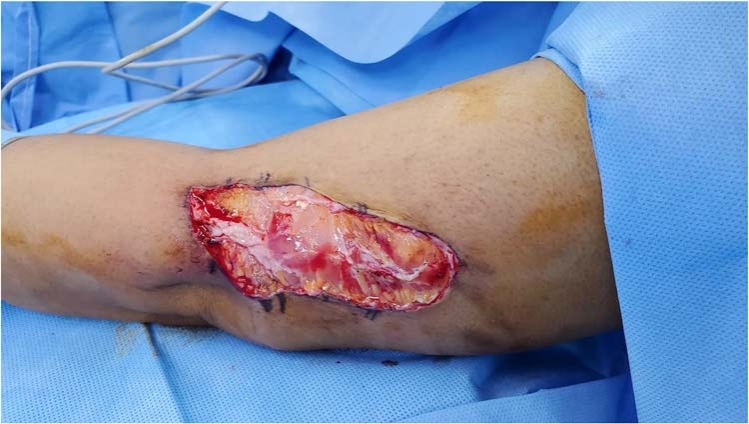
Wound post debridement.

**Figure 3:**
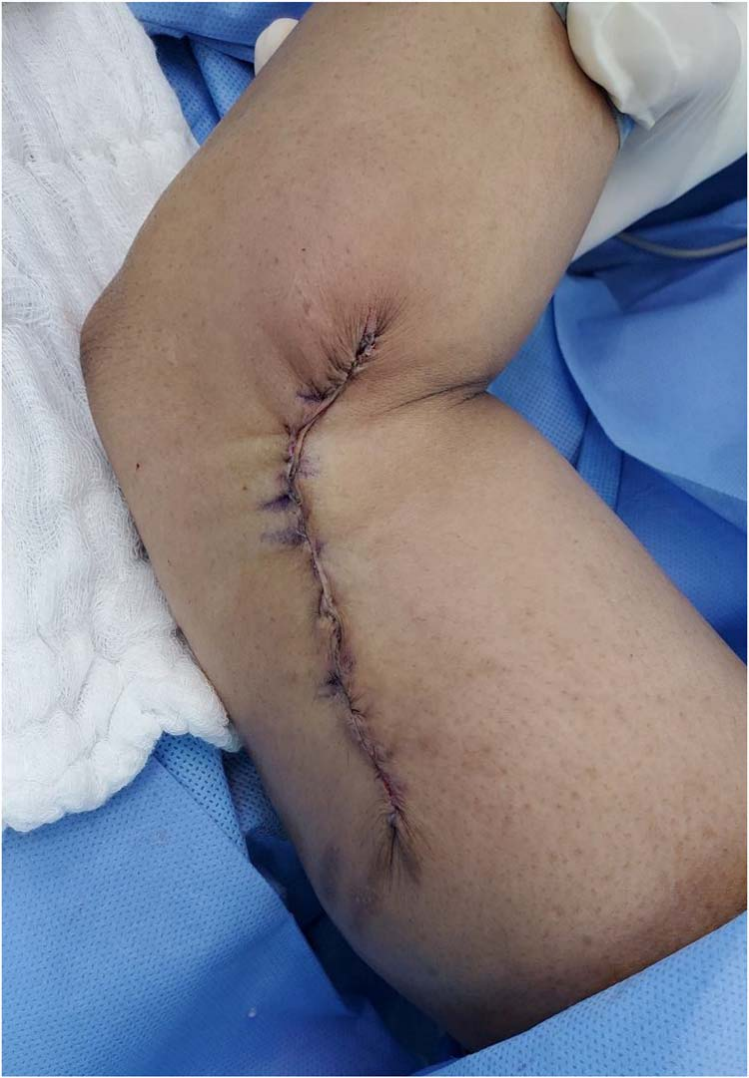
The wound post primary closur.

The wound healed well but exhibited widening and hypertrophic scarring. After the wound healed, scar care was initiated with silicon sheets, laser therapy, and steroid injections (Figs 2 and 3).

## DISCUSSION

After a thorough literature review, to the best of our knowledge, skin necrosis has not been reported as a complication following pethidine administration. Accordingly, we also determined if there was an indirect complication related to skin necrosis.

The most common diagnosis that comes to mind for skin necrosis is an extravasation injury, which is the accumulation of fluid outside the vein. This causes pressure and irritation to the tissue, which may lead to local inflammation, skin necrosis, and compartment syndrome. In our case, the site of the intravenous line was the dorsum of the hand. According to the patient’s history, it was negative for swelling, and the two lesions in the thigh and ear did not demonstrate extravasation [7].

Another possible diagnosis is Nicolau Syndrome, which is a rare complication that can happen after an intramuscular injection, causing skin necrosis. Its pathogenesis is not completely known but is presumed to be induced by direct vascular injury, inflammation, and vascular constriction. Our patient did not receive medication intramuscularly; therefore, this diagnosis was less likely [8].

Steven-Johnson Syndrome (SJS) and toxic epidermal necrolysis (TEN) are acute mucocutaneous syndromes that occur as a result of immune reactions to certain medications and are characterised by the separation of the epidermis from the dermis. They can also be caused by infections, vaccines, or idiopathic diseases. The difference between these two diseases is the extent of the detached skin surface area. However, the patient notably had no history of fever, upper respiratory tract symptoms, or mucosal ulceration before or during the development of the skin lesions. She had been using paracetamol since childhood without any adverse reactions. In addition, the total surface area of the wound is approximately 1% of the total body surface area and spans full thickness [9].

Warfarin, heparin, and anticoagulants can cause skin necrosis in rare cases. It occurs when warfarin rapidly depletes vitamin K-dependent coagulation factors, leading to localised thrombosis and subsequent skin necrosis. Meanwhile, heparin may cause an immune-mediated hypersensitivity reaction or localised thrombosis. Risk factors for warfarin-induced skin necrosis include high initial warfarin dose, female sex, obesity, and certain genetic factors, while risk factors for heparin-induced skin necrosis include female sex, obesity, and underlying coagulopathies; however, the patient is not receiving any anticoagulant medication [10].

In the literature, there was a case of an unusual reaction to intravenous pethidine, in which redness over the vein receiving pethidine settled after normal saline was flushed for 15 minutes. However, there was no mention of any subsequent skin changes or necrosis [11].

As discussed, our patient had no clear diagnosis for skin necrosis, did not receive any medication except pethidine, and had no history of allergy, raising the question of whether pethidine can cause skin necrosis. To the best of our knowledge, this is the first reported case in the literature for pethidine-induced skin necrosis, thus requiring more attention if more unreported cases are occurring worldwide.

## Data Availability

The database set related to the manuscript submitted is available upon request.
